# Sensory Neurons, PIEZO Channels and PAC1 Receptors Regulate the Mechanosensitive Release of Soluble Ectonucleotidases in the Murine Urinary Bladder Lamina Propria

**DOI:** 10.3390/ijms24087322

**Published:** 2023-04-15

**Authors:** Mafalda S. L. Aresta Branco, Alejandro Gutierrez Cruz, Mahsa Borhani Peikani, Violeta N. Mutafova-Yambolieva

**Affiliations:** Department of Physiology and Cell Biology, School of Medicine, University of Nevada, Reno, NV 89557, USA

**Keywords:** ATP, urothelium, ectonucleotidases, PIEZO, PAC1 receptor, sensory neuropeptides, PACAP, bladder, purinergic, bladder lamina propria

## Abstract

The urinary bladder requires adequate concentrations of extracellular adenosine 5′-triphosphate (ATP) and other purines at receptor sites to function properly. Sequential dephosphorylation of ATP to ADP, AMP and adenosine (ADO) by membrane-bound and soluble ectonucleotidases (s-ENTDs) is essential for achieving suitable extracellular levels of purine mediators. S-ENTDs, in particular, are released in the bladder suburothelium/lamina propria (LP) in a mechanosensitive manner. Using 1,N^6^-etheno-ATP (eATP) as substrate and sensitive HPLC-FLD methodology, we evaluated the degradation of eATP to eADP, eAMP and eADO in solutions that were in contact with the LP of ex vivo mouse detrusor-free bladders during filling prior to substrate addition. The inhibition of neural activity with tetrodotoxin and ω-conotoxin GVIA, of PIEZO channels with GsMTx4 and D-GsMTx4 and of the pituitary adenylate cyclase-activating polypeptide type I receptor (PAC1) with PACAP6-38 all increased the distention-induced but not spontaneous release of s-ENTDs in LP. It is conceivable, therefore, that the activation of these mechanisms in response to distention restricts the further release of s-ENTDs and prevents excessive hydrolysis of ATP. Together, these data suggest that afferent neurons, PIEZO channels, PAC1 receptors and s-ENTDs form a system that operates a highly regulated homeostatic mechanism to maintain proper extracellular purine concentrations in the LP and ensure normal bladder excitability during bladder filling.

## 1. Introduction

The main functions of the bladder, storing and eliminating urine, are regulated by complex neural pathways that are triggered by the distention (stretching) of the bladder wall during the filling of the bladder with urine. The urothelium, a frontline sensor in the bladder, contributes to mechanotransduction by releasing chemical mediators in response to stretching that activate neural pathways initiating micturition. Adenosine 5′-triphosphate (ATP) is considered to be a key factor in the interactions between urothelial cells and sensory neurons to control urination [1]. In particular, it was demonstrated that stretching induces the release of ATP from isolated bladder mucosa sheets pinned in Ussing chambers [2] and that mice lacking *P2rx2* and *P2rx3* exhibit signs of bladder underactivity [3,4]. These observations, which were made about two decades ago, sparked tremendous interest in purinergic signaling in the lower urinary tract that has not diminished to this day. It was hypothesized that ATP is released from the urothelium into the suburothelium/lamina propria (LP), where it activates afferent neurons and stimulates voiding [3,4,5,6]. In the following years, it became clear that a broad class of purinergic receptors, including ligand-gated ion channel P2X receptors, G-protein coupled P2Y receptors and G-protein-coupled adenosine receptors, is expressed in all major cell types in the bladder mucosa [1,7]. Therefore, extracellular ATP may contribute to sensory processing through the activation of purinergic receptors on various cell types within the bladder wall, in addition to sensory afferents. To perform its functions, however, ATP must be in effective concentrations in the vicinity of its targeted receptors.

It is widely recognized that extracellular levels of ATP are regulated by enzymes located at the cell surface, commonly referred to as ectonucleotidases [8]. ATP hydrolysis by ectonucleotidases results in diminished ATP concentrations and increased concentrations of its metabolites, namely adenosine 5′-diphosphate (ADP), adenosine 5′-monophosphate (AMP) and adenosine (ADO), in proximity to purinergic receptors. Therefore, ectonucleotidases have an immense impact on purinergic receptor signaling. We recently discovered that the extracellular hydrolysis of ATP in the bladder LP occurs in a mechanosensitive manner so that the degradation of ATP in LP is increased when the bladder wall is gradually distended by the filling of the bladder [9]. This seems to be counterintuitive, given the presumed role of ATP in initiating voiding. It is likely, however, that ATP has additional functions since ATP is also released from the bladder mucosa at low filling volumes when no voiding occurs [10,11]. Because of the multifaceted biological activity of ATP, understanding the mechanisms that control its availability at receptor sites is of utmost significance. It is important to note that, in addition to membrane-bound ectonucleotidases (mb-ENTDs), soluble ectonucleotidases (s-ENTDs) are released/secreted in the bladder LP and contribute meaningfully to the mechanosensitive extracellular hydrolysis of ATP [9]. s-ENTDs have the potential to be better therapeutic targets than mb-ENTDs because s-ENTDs likely have specialized cell sources and specific mechanisms of release in the extracellular space [8,12], whereas mb-ENTDs are confined to the membranes of virtually all cell types [8], which makes them a challenging target. Likewise, s-ENTDs might represent better targets to manipulate the effective concentrations of purines at receptor sites than the numerous mechanisms of the vesicular and non-vesicular release of ATP or the large number of ubiquitously expressed purinergic receptors. However, more work needs to be done to understand the mechanisms that control the mechanosensitive release of s-ENTDs in the bladder wall and in LP, in particular.

The stretching of the bladder wall during bladder filling elicits a plethora of physiological responses mediated by various signaling pathways. Thus, stretching and changes in pressure during bladder filling are detected by the urothelium [13,14] and by mechanically sensitive afferent neurons within the urothelium and LP that are presumed to be sensitive to tetrodotoxin but insensitive to capsaicin [15,16,17,18]. Stretching also activates PIEZO proteins—mechanically gated ion channels expressed in various cell types, including cells in the urothelium and LP and in innervating sensory neurons [19,20]. The mechanosensitivity of afferent neurons in the bladder wall is modulated by endogenous neuropeptides such as the pituitary adenylate cyclase activator peptide (PACAP) [21] and neurokinin A (NKA) [22]. While studies in different systems have suggested links between stretching, the activation of PIEZO channels, the activation of neuropeptide receptors and ATP efflux [22,23,24,25], it is currently unknown whether such mechanisms regulate the mechanosensitive release of s-ENTDs and, hence, the degradation of ATP in the bladder LP. Therefore, the present study was designed to examine the possible interplay between sensory neurons, PIEZO channels and endogenous PACAP38 and NKA in the regulation of the constitutive and distention-induced release of s-ENTDs in the bladder LP.

## 2. Results

### 2.1. Capsaicin Does Not Alter the Spontaneous and the Distention-Induced Release of s-ENTDs in LP

**Figure 1:**
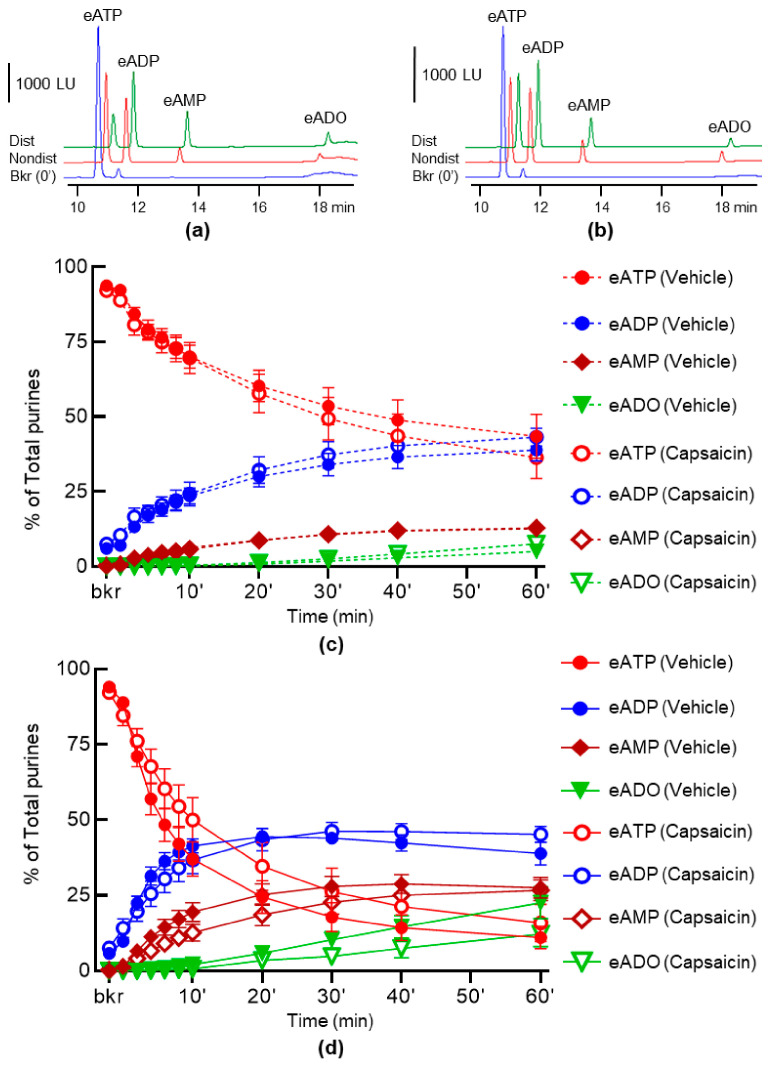
**Effect of capsaicin on the hydrolysis of eATP by s-ENTDs released in cELS of detrusor-free bladder preparations.** Original chromatograms of eATP in beaker (Bkr, blue, 0′, no enzyme present) and at 60 min of enzymatic reaction in cELS of nondistended (Nondist, red) and distended (Dist, green) bladder preparations treated with vehicle (**a**) or capsaicin (**b**). LU, luminescence units. Note the decrease of eATP substrate and the increase or appearance of the 1,N^6^-etheno-products (e-products) 1,N^6^-etheno-ADP (eADP), 1,N^6^-etheno-AMP (eAMP) and 1,N^6^-etheno-ADO (eADO). Summarized data showing time-courses of eATP hydrolysis in cELS in the presence of vehicle (DMSO 0.1%, *n* = 7) or capsaicin 1 µM (*n* = 5) in nondistended (**c**) and distended (**d**) bladders; *n*, number of bladder preparations. eATP, eADP, eAMP and eADO are presented as percentages of total purines (eATP + eADP + eAMP + eADO) present in reaction solutions. *p* > 0.05, vehicle control vs. capsaicin, two-way ANOVA with Sidak’s multiple comparisons tests.

To assess the effect of activation of capsaicin-sensitive afferent neurons on the release of s-ENTDs, we evaluated the 1,N^6^-etheno-ATP (eATP) hydrolysis in concentrated extraluminal solutions (cELS) (see Section 4.3, Section 4.4 and Section 4.5) of nondistended and distended bladder preparations treated with capsaicin. As shown in Figure 1, capsaicin failed to change the spontaneous and the distention-induced release of s-ENTDs in the LP of detrusor-free bladder preparations, suggesting that the release of s-ENTDs is largely capsaicin-insensitive. Additionally, note that in the vehicle controls, the distention-induced release of s-ENTDs exceeded the spontaneous release of s-ENTDs (Figure 1a,c,d), in confirmation of our previous results [9].

### 2.2. Neural Blockade with Tetrodotoxin (TTX) Has No Effect on the Spontaneous Release of s-ENTDs but Increases the Distention-Induced Release of s-ENTDs in LP

**Figure 2:**
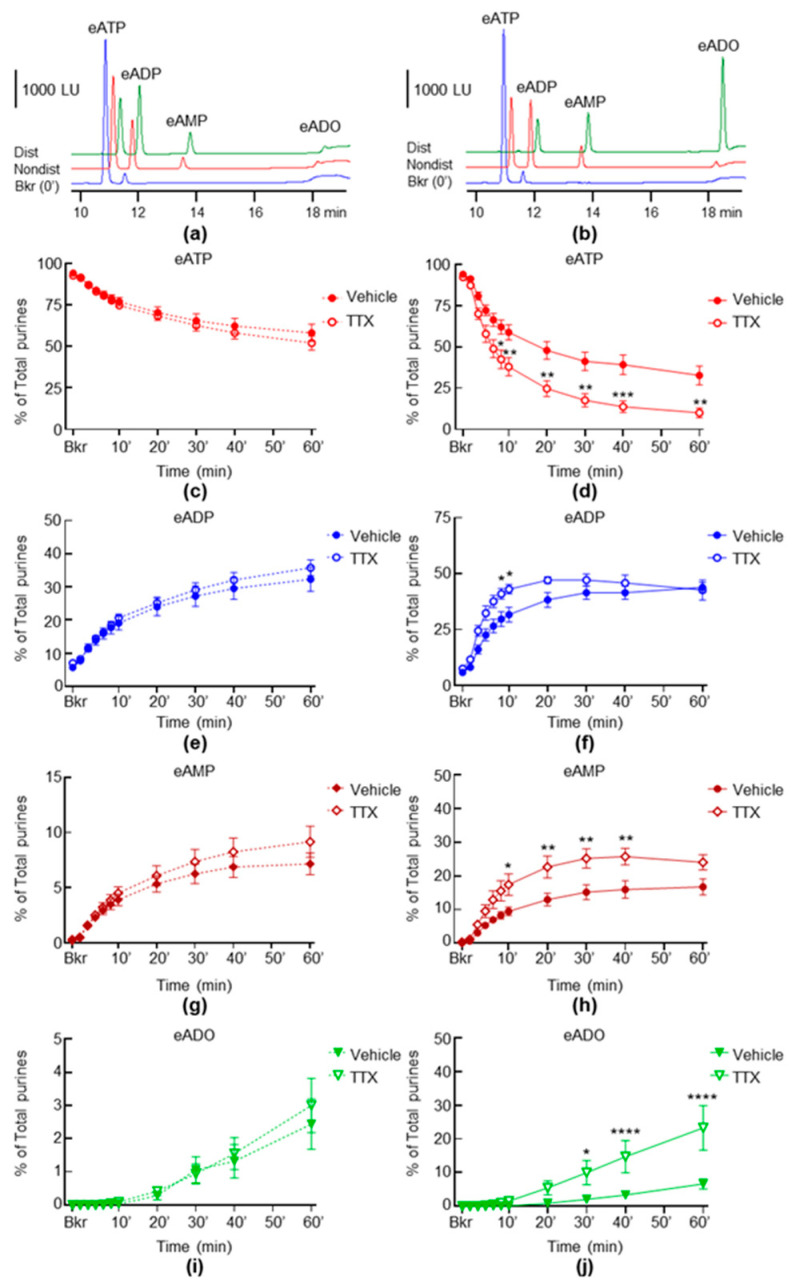
**Effects of TTX on the hydrolysis of eATP by s-ENTDs released in cELS of nondistended and distended detrusor-free bladder preparations.** Original chromatograms of eATP in beaker (Bkr, blue, 0′, no enzyme present) and at 60 min of enzymatic reaction in cELS of nondistended (Nondist, red) and distended (Dist, green) bladder preparations treated with vehicle (**a**) or TTX (**b**). LU, luminescence units. Summarized data showing changes in eATP, eADP, eAMP and eADO in the presence of vehicle (Krebs bicarbonate solution, KBS, *n* = 8) or TTX 0.5 µM (*n* = 6) in nondistended (**c**,**e**,**g**,**i**) and distended (**d**,**f**,**h**,**j**) bladder preparations; *n*, number of bladder preparations. eATP, eADP, eAMP and eADO are presented as percentages of total purines (eATP + eADP + eAMP + eADO) present in reaction solutions. Asterisks denote significant difference from vehicle controls. * *p* < 0.05, ** *p* < 0.01, *** *p* < 0.001, **** *p* < 0.0001. Two-way ANOVA with Sidak’s multiple comparisons tests.

To determine the effect of neural inhibition on the release of s-ENTDs, we evaluated the eATP hydrolysis in cELS of nondistended and distended bladder preparations treated with TTX. As shown in Figure 2, the incubation of cELS of both nondistended (Figure 2c,g,i) and distended (Figure 2d,f,h,j) bladder preparations with eATP resulted in the gradual decrease of eATP (Figure 2c,d) and increase in eADP (Figure 2e,f), eAMP (Figure 2g,h) and eADO (Figure 2i,j). The eATP hydrolysis in cELS of distended preparations exceeded the eATP hydrolysis from nondistended preparations. The degradation of eATP was similar in cELS of the control and TTX-treated nondistended preparations (Figure 2c,g,i). In contrast, the hydrolysis of eATP in cELS of distended preparations was significantly increased in the presence of TTX (Figure 2d,f,h,j). Thus, the eATP decrease in TTX-treated samples was significantly accelerated shortly after the initiation of the reaction (Figure 2d). The half-life of eATP (e.g., 50% reduction) was approximately 20 min in cELS of untreated preparations and 6 min in cELS of TTX-treated preparations. Consequently, the eATP products eADP, eAMP and eADO were significantly increased in the presence of TTX, suggesting that the inhibition of action potential firings/propagation with TTX facilitated the distention-induced release of s-ENTDs in the LP.

### 2.3. Neural Blockade with ω-Conotoxin (ω-CtxGVIA) Increases Both the Spontaneous and the Distention-Induced Release of sNTDs

**Figure 3:**
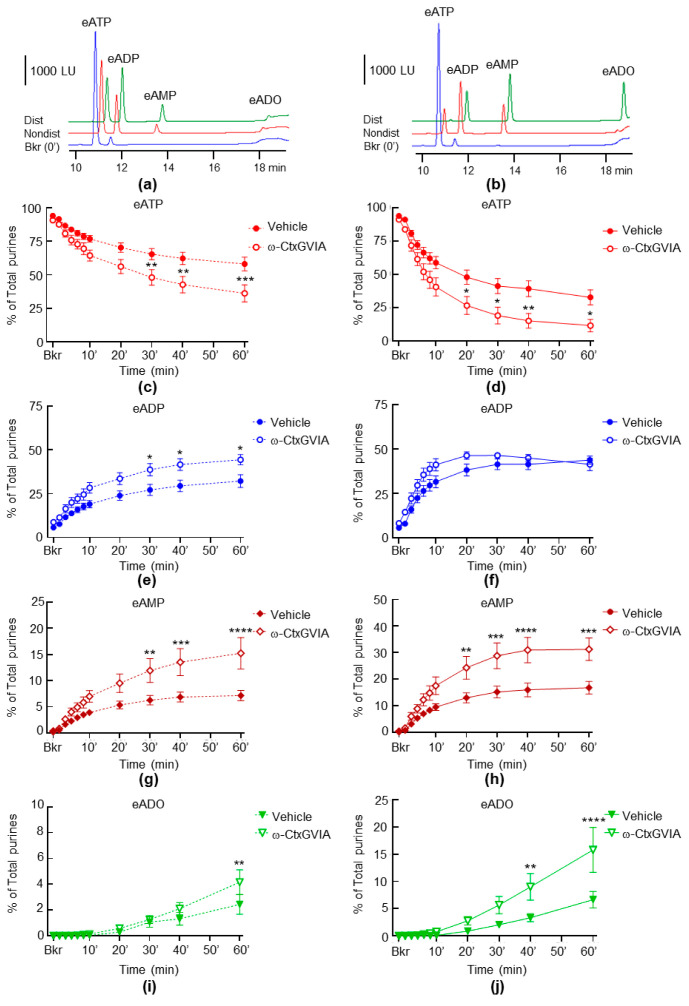
**Effects of ω-Ctx GVIA on the hydrolysis of eATP by s-ENTDs released in cELS of nondistended and distended detrusor-free bladder preparations.** Original chromatograms of eATP in beaker (Bkr, blue, 0′, no enzyme present) and at 60 min of enzymatic reaction in cELS of nondistended (Nondist, red) and distended (Dist, green) bladder preparations treated with vehicle (**a**) or ω-Ctx GVIA (**b**). LU, luminescence units. Summarized data showing changes in eATP, eATP, eADP, eAMP and eADO in the presence of vehicle (KBS, *n* = 8) and ω-CtxGVIA 0.1 µM (*n* = 6) in nondistended (**c**,**e**,**g**,**i**) and distended (**d**,**f**,**h**,**j**) bladder preparations; *n*, number of bladder preparations. eATP, eADP, eAMP and eADO are presented as percentages of total purines (eATP + eADP + eAMP + eADO) present in reaction solutions. Asterisks denote significant difference from vehicle controls. * *p* < 0.05, ** *p* < 0.01, *** *p* < 0.001, **** *p* < 0.0001. Two-way ANOVA with Sidak’s multiple comparisons tests.

We next investigated whether a neural blockade with the inhibitor of neural voltage-dependent Ca_v_2.2 channels (also known as N-type Ca^2+^ channels) ω-Ctx GVIA would alter the release of s-ENTDs in LP. Incubation with ω-Ctx GVIA resulted in a significantly greater eATP decrease and e-product increase in cELS collected from both nondistended (Figure 3c,e,g,i) and distended (Figure 3d,f,h,j) preparations.

### 2.4. Inhibition of PIEZO Channels Has No Effect on the Spontaneous Release of s-ENTDs, but Increases the Distention-Induced Release of s-ENTDs in LP

**Figure 4:**
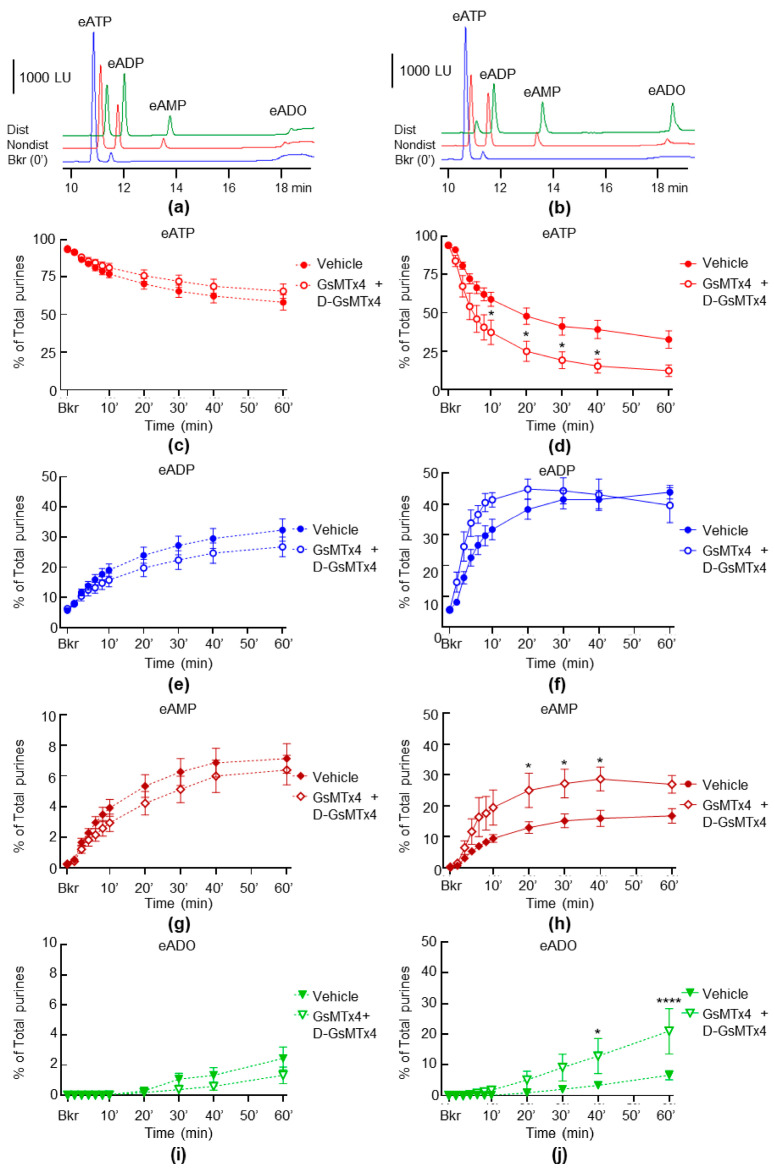
**Effects of GsMTx4 plus D-GsMTx4 on the hydrolysis of eATP by s-ENTDs released in cELS of nondistended and distended detrusor-free bladder preparations.** Original chromatograms of eATP in beaker (Bkr, blue, 0′, no enzyme present) and at 60 min of enzymatic reaction in cELS of nondistended (Nondist, red) and distended (Dist, green) bladder preparations treated with vehicle (**a**) or GsMTx4 plus D-GsMTx4 (**b**). LU, luminescence units. Summarized data showing changes in eATP, eATP, eADP, eAMP and eADO in the presence of vehicle (KBS, *n* = 8) and GsMTx4+D-GsMTx4, 1 µM each (*n* = 6) in nondistended (**c**,**e**,**g**,**i**) and distended (**d**,**f**,**h**,**j**) bladder preparations; *n*, number of bladder preparations. eATP, eADP, eAMP and eADO are presented as percentages of total purines (eATP + eADP + eAMP + eADO) present in reaction solutions. Asterisks denote significant difference from vehicle controls. * *p* < 0.05, **** *p* < 0.0001. Two-way ANOVA with Sidak’s multiple comparisons tests.

**Figure 5:**
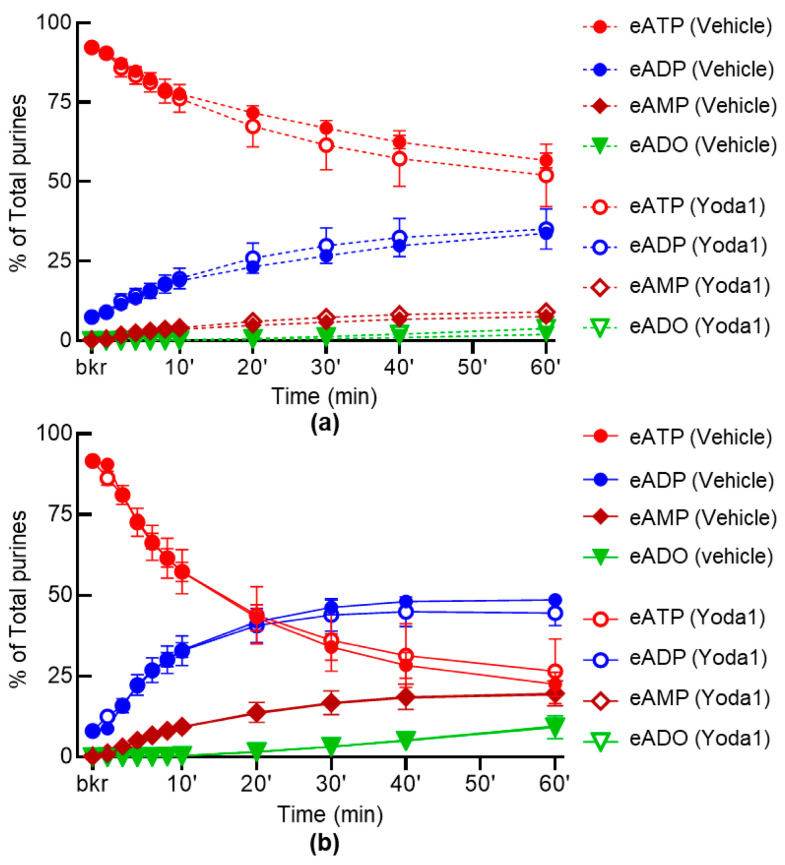
**Effect of YODA1 on the hydrolysis of eATP by s-ENTDs released in cELS of detrusor-free bladder preparations.** Changes in eATP, eADP, eAMP and eADO in the presence of vehicle (DMSO 0.2%, *n* = 6) or Yoda1, 40 µM (*n* = 5) in nondistended (**a**) and distended (**b**) bladder preparations; *n*, number of bladder preparations. eATP, eADP, eAMP and eADO are presented as percentages of total purines (eATP + eADP + eAMP + eADO) present in reaction solutions. *p* > 0.05 at all time-points of reaction. Two-way ANOVA with Sidak’s multiple comparisons tests.

To study whether the inhibition of PIEZO channels has an effect on the release of s-ENTDs, we evaluated the hydrolysis of eATP in cELS of nondistended and distended bladder preparations treated with GsMTx4 and D-GsMTx4. As shown in Figure 4, in the presence of the combined PIEZO channel inhibitors, there was a significantly greater decrease in eATP and increase in eADP, eAMP and eADO in the cELS of distended preparations (Figure 4d,f,h,j). In contrast, the decrease in eATP and increase in e-products were unaffected by GsMtx4 plus D-GsMTx4 in cELS of nondistended preparations (Figure 4c,e,g,i). Of note, GsMTx4 and D-GsMTx4 alone had no significant effect on the degradation of eATP neither in cELS of nondistended nor of distended bladders (data not shown). The PIEZO1 activator Yoda1 had no significant effect on either the spontaneous or distention-induced release of s-ENTDs (Figure 5a,b).

### 2.5. Inhibition of TRPC1 and TRPC6 Channels Have Weak Effect on the Spontaneous and Distention-Induced Release of s-ENTDs in LP

**Figure 6:**
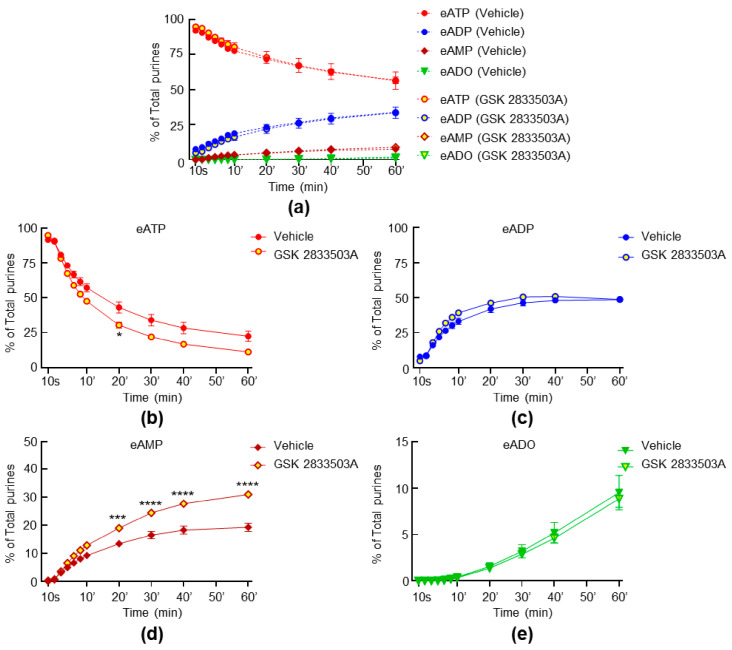
**Effects of GSK 2833503A on the hydrolysis of eATP by s-ENTDs released in cELS of detrusor-free bladder preparations.** Changes in eATP, eADP, eAMP and eADO in the presence of vehicle (DMSO 0.2%, *n* = 6) and GSK 2833503A, 1 µM (*n* = 3) in nondistended (**a**) and distended (**b,c,d,e**) bladder preparations; *n*, number of bladder preparations. eATP, eADP, eAMP and eADO are presented as percentages of total purines (eATP + eADP + eAMP + eADO) present in reaction solutions. Asterisks denote significant difference from vehicle controls. * *p* < 0.05, *** *p* < 0.001, **** *p* < 0.0001. Two-way ANOVA with Sidak’s multiple comparisons tests.

Although GsMTx4 has a high potency for PIEZO channels, it has also been suggested to block transient receptor potential cation TRPC1 and TRPC6 channels. Therefore, we next investigated whether TRPC1 or TRPC6 might be involved in the regulation of s-ENTD release. Neither the spontaneous nor the distention-induced release of s-ENTDs in the LP was affected by the potent inhibitor of the TRPC1/4/5 channel Pico145 and the TRPC6/3 channel activator GSK 1702934A (data not shown). Likewise, the TRPC6/3 channel inhibitor GSK 2833503A had no effect on the spontaneous release of s-ENTDs (Figure 6a). However, the distention-induced degradation of eATP showed a tendency to be increased, reaching statistical significance only at 20 min of reaction (Figure 6b). The formation of eADP (Figure 6c) or eADO (Figure 6e) was not altered in the presence of GSK 283503A, whereas the levels of eAMP were elevated at 20–60 min of reaction (Figure 6d).

**Figure 7:**
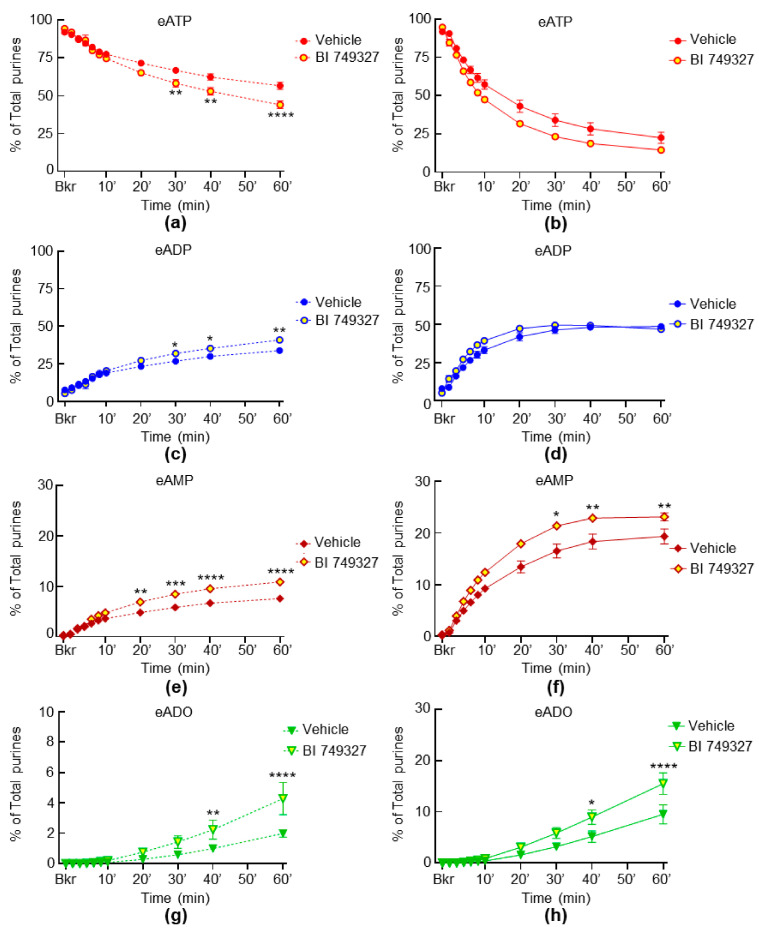
**Effects of BI 74932 on the hydrolysis of eATP by s-ENTDs released in cELS of nondistended and distended detrusor-free bladder preparations.** Changes in eATP, eADP, eAMP and eADO in the presence of vehicle (DMSO 0.2%, *n* = 6) and BI 74932, 1 µM (*n* = 3) in nondistended (**a**,**c**,**e**,**g**) and distended (**b**,**d**,**f**,**h**) bladder preparations; *n*, number of bladder preparations. eATP, eADP, eAMP and eADO are presented as percentages of total purines (eATP + eADP + eAMP + eADO) present in reaction solutions. Asterisks denote significant difference from vehicle controls. * *p* < 0.05, ** *p* < 0.01, *** *p* < 0.001, **** *p* < 0.0001. Two-way ANOVA with Sidak’s multiple comparisons tests.

The spontaneous degradation of eATP was increased in the presence of the specific TRPC6 channel inhibitor BI 749327, resulting in the increased formation of eADP, eAMP and eADO at some time points of enzymatic reaction in the cELS of nondistended preparations (Figure 7c,e,g). The decrease of eATP and increase of eADP in response to distention were not affected by BI 749327 (Figure 7b,d), but the formation of eAMP and eADO was increased (Figure 7f,h).

### 2.6. Inhibition of Receptors for Sensory Neuropeptides Affects Differentially the Release of s-ENTDs in LP

**Figure 8:**
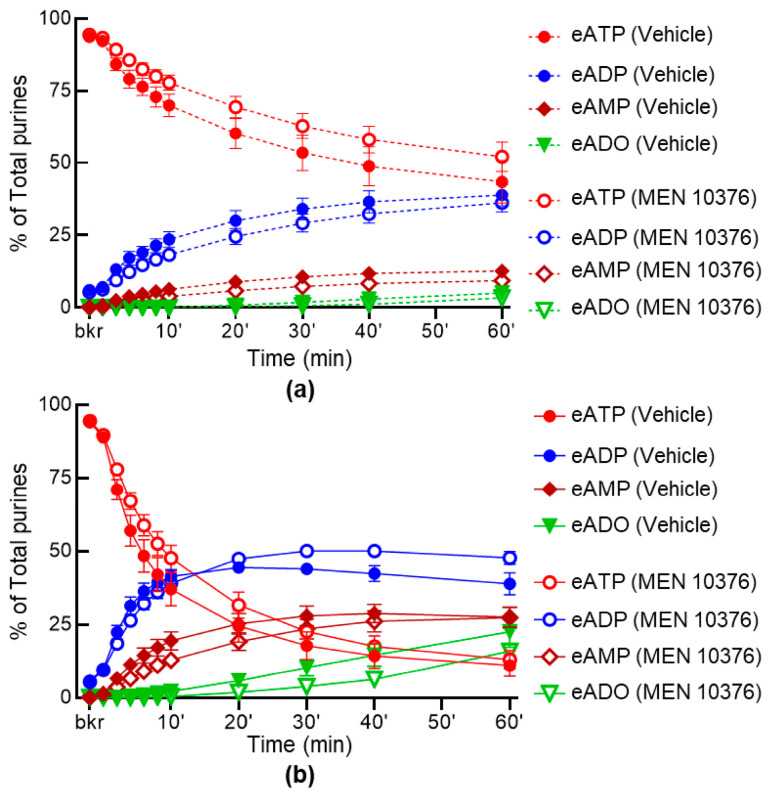
**Effect of MEN 10376 on the hydrolysis of eATP by s-ENTDs released in cELS of detrusor-free bladder preparations.** Changes in eATP, eADP, eAMP and eADO in the presence of vehicle (DMSO 0.1%, *n* = 7) in nondistended (**a**) and distended (**b**) bladder preparations in the presence of MEN 10376, 10 µM (*n* = 4); *n*, number of bladder preparations. eATP, eADP, eAMP and eADO are presented as percentages of total purines (eATP + eADP + eAMP + eADO) present in reaction solutions. *p* > 0.05 at all time-points of reaction in the presence of drug. Two-way ANOVA with Sidak’s multiple comparisons tests.

**Figure 9:**
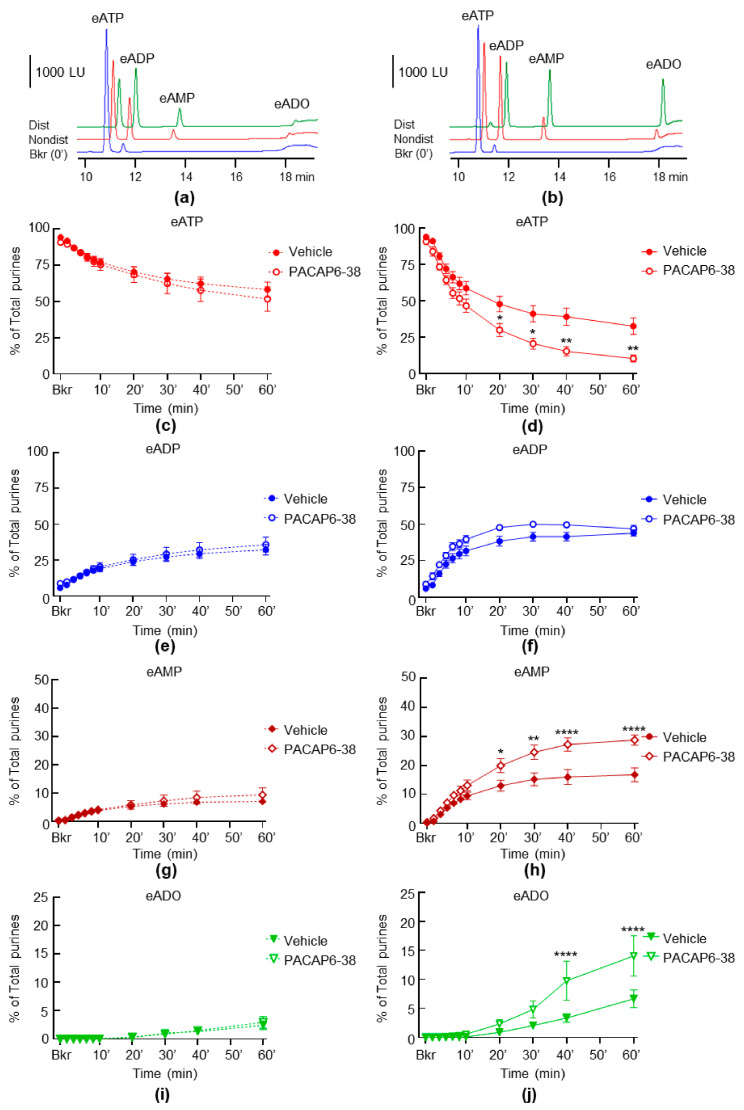
**Effects of PACAP6-38 on the hydrolysis of eATP by s-ENTDs released in cELS of nondistended and distended detrusor-free bladder preparations.** Original chromatograms of eATP in beaker (Bkr, blue, 0′, no enzyme present) and at 60 min of enzymatic reaction in cELS of nondistended (Nondist, red) and distended (Dist, green) bladder preparations treated with vehicle (**a**) or PACAP6-38 (**b**). LU, luminescence units. Summarized data showing changes in eATP, eATP, eADP, eAMP and eADO in the presence of vehicle (KBS, *n* = 8) and PACAP6-38, 0.3 µM (*n* = 5) in nondistended (**c**,**e**,**g**,**i**) and distended (**d**,**f**,**h**,**j**) bladder preparations; *n*, number of bladder preparations. eATP, eADP, eAMP and eADO are presented as percentages of total purines (eATP + eADP + eAMP + eADO) present in reaction solutions. Asterisks denote significant difference from vehicle controls. * *p* < 0.05, ** *p* < 0.01, **** *p* < 0.0001. Two-way ANOVA with Sidak’s multiple comparisons tests.

We next investigated whether selective antagonists of NK2 and PAC1 receptors influence the release of s-ENTDs in LP. Neither the spontaneous nor the distention-induced release of s-ENTDs was altered in the presence of the NK2 receptor antagonist MEN 10376 (Figure 8a,b). However, a greater degradation of eATP was observed in the presence of the PAC1 receptor antagonist PACAP6-38 in the cELS of distended preparations (Figure 9). Thus, the decrease of eATP and the increase of eAMP were significantly greater at 20–30 min of reaction (Figure 9d,h), whereas the eADO increase reached significance at 40–60 min of reaction (Figure 9j).

## 3. Discussion

To function properly, the urinary bladder must refrain from premature contraction during filling and emptying when critical intravesical volume and pressure are reached. It is generally presumed that (i) distention of the bladder wall during filling increases sensory nerve activity [26,27], (ii) the bladder mucosa secretes ATP in response to physical and chemical stimuli [2,23,28] and (iii) the activation of afferent nerves in LP and urothelium by extracellular ATP initiates voiding [1]. Therefore, ATP is believed to play a significant sensory role in the control of normal micturition [29]. In addition, bladder excitability disorders may also be associated with aberrant purinergic signaling since increased intraluminal levels of ATP are found in patients with overactive bladder syndrome [10,30], interstitial cystitis and bladder pain syndrome [31,32]. The potential roles of ATP in bladder physiology and pathophysiology makes it important to understand the mechanisms that control the ATP concentrations at receptor sites.

Extracellular ATP concentrations are determined by two processes that occur almost simultaneously—ATP release and ATP removal [33]. However, changes in extracellular ATP levels are commonly considered to represent direct changes in ATP release, disregarding the effect ATP metabolism might have on measured ATP levels. Moreover, when ATP hydrolysis is considered, it is commonly regarded as means for ATP removal, overlooking the fact that ATP degradation could guarantee the biological activity of purine mediators by regulating their effective concentrations at receptor sites. In the urinary bladder, ATP is metabolized to ADP, AMP and ADO [11,34,35] by canonical ENTDs that are bound to cell membranes [8], as well as by s-ENTDs that are released in the LP and in bladder lumen [9,36]. At the end of bladder filling, ATP represents only about 5% of the total purine pool (i.e., ATP+ADP+AMP+ADO), whereas ADO is the dominant purine present in the LP [34]. In the present study, we investigated the potential role of sensory neurons and mechanosensitive ion channels on the release of s-ENTDs in the LP and, consequently, on the degradation of extracellular ATP. We found that the distention-induced but not the spontaneous release of s-ENTDs in the LP is increased via the inhibition of neural activity and of mechanosensitive ion channels. These findings were unexpected, as they suggested, together with our previous observations [9], that the distention of the bladder wall during physiologic filling activates mechanisms that have opposite effects—increasing and restraining the release of s-ENTDs in response to bladder wall distention. It appears, therefore, that the extracellular levels of ATP and other purine mediators deep in the bladder wall are under more complex regulation than we originally thought.

To eliminate the potential influence of the CNS, the systemic circulation or the detrusor muscle, and to obtain direct access to the LP at rest or during bladder filling, we carried out the study in an ex vivo murine bladder model with intact bladder mucosa but no detrusor [9,34,36]. This model is superior to commonly used bladder wall strips, bladder mucosa sheets and cultured urothelial cells because it enables access to LP while replicating the forces that layers of the bladder wall experience during bladder filling. Reproducing authentic bladder filling in studies of mechanosensitive mechanotransduction in the bladder wall is important because the peripheral termini of sensory neurons innervating the bladder are equipped to detect a variety of mechanical stimuli, including 3D-distention and the circumferential stretching of the organ. Moreover, a recent study using three-dimensional nerve tracking by two-photon laser scanning microscopy confirmed that the highest concentration of afferent nerve fibers in the bladder wall is at the urothelium-LP interface [37]. Therefore, having direct access to this layer of the bladder wall in the course of bladder filling is essential for studies of sensory mechanotransduction. Many studies measure the intraluminal content of ATP and assume that it reflects the ATP content on the abluminal side of the urothelium. However, such studies are highly inaccurate since luminal fluid reflects only the release of mediators by umbrella cells and not the basal or intermediate urothelial cells or cells in the LP that are in a close apposition to nerve endings. The detrusor-free bladder model contains the majority of interstitial cells that reside in the LP, suggesting that the LP is largely preserved in this preparation [34]. The initial validation of the model demonstrated that the pressure–volume relationships during physiological bladder filling were similar in ex vivo bladder preparations with intact and removed detrusor smooth muscle [34]. Furthermore, a lactate dehydrogenase cell toxicity assay of cELS demonstrated that the detrusor-free bladder preparation does not display greater cell damage than the preparation with intact smooth muscle [9]. Finally, the bladder filling rate of 15 µL/min; 0.9 mL/h, which was used in the present study and in our previous work, is estimated to be within the normal physiological bladder filling rates in mice [38], reinforcing the physiological relevance of the model. Overall, the detrusor-free bladder model is suitable for studies that are focused on the local mechanisms of sensory innervation of the LP and urothelium, where micturition reflexes that govern urination are initiated.

As previously, we used 1,N^6^-etheno-ATP (eATP, 2 µM) as a substrate and measured the decrease of eATP and the increase of its metabolites, eADP, eAMP and eADO, over 1 h of contact between cELS and eATP [9,34,36]. Using an eATP substrate instead of ATP has significant advantages [9]. Thus, (i) the use of eATP has been validated as an effective technique to assess the extracellular metabolism of ATP [39], (ii) the method detects e-purines with superb sensitivity (i.e., femtomolar range) and (iii) the possible release of endogenous purine mediators remains undetected, suggesting that changes in eATP and e-products reflects solely the degradation of ATP.

Using the detrusor-free preparation and eATP substrate, we previously found that ATP is degraded to ADP, AMP and ADO in the LP of both empty/nondistended and full/distended bladders, and the degradation of ATP is greater in distended than in nondistended LP [9,34]. We also determined that several enzymes with characteristics of ectonucleoside triphosphate diphosphohydrolases (e.g., ENTPD1,3,8), ectonucleotide pyrophosphatase/phosphodiasterases (e.g., ENPP3), and 5′-nucleotidase (NT5E) are secreted/released in the LP during bladder filling in a regulated manner [9,36]. These enzymes constitute the pool of s-ENTDs in the LP and provide means for the precise control of extracellular levels of purine mediators.

Since stretching/distention activates sensory afferent nerves in the bladder wall [15] and increases the release of s-ENTDs in the LP [9], our goal in the present study was to determine whether there is a link between neural activity and the release of ATP-degrading enzymes in the LP. We hypothesized that, if the distention-induced release of s-ENTDs depends on the activation of sensory neurons in response to distention, then a neural blockade should inhibit the distention-induced release of s-ENTDs but should not alter the spontaneous release of s-ENTDs. Pelvic nerve afferents that innervate the bladder are generally divided into two populations: small myelinated Aδ and unmyelinated C-fibers. Myelinated Aδ-fiber afferents are silent when the bladder is empty but are gradually activated during bladder filling and trigger the normal micturition reflex [18]. In contrast, the C-fiber bladder afferents are considered to be mostly mechano-insensitive but could become mechanosensitive following exposure to chemical irritants and cold [18,40,41]. Based on their sensitivity to neurochemicals, bladder afferent neurons are divided broadly into two populations: (1) sensitive to capsaicin and insensitive to tetrodotoxin and (2) insensitive to capsaicin and sensitive to TTX [18]. The capsaicin-sensitive neurons are assumed to be the origin of C-fiber afferents that are generally mechano-insensitive, whereas TTX-sensitive responses appear to be more typical for Aδ-fiber afferent neurons [15,18]. TTX-sensitive Na_v_ channels, in particular, regulate the neural excitability of bladder dorsal root ganglia (DRG) neurons and mediate almost all bladder afferent responses to distention [42]. In the present study, capsaicin failed to affect the release of s-ENTDs in both nondistended and distended preparations, suggesting that the activation of capsaicin-sensitive C-fiber afferents in the LP may not be the primary mechanism for the control of s-ENTD release in the bladder LP. In the presence of TTX, however, the spontaneous release of s-ENTDs remained unaltered, while the distention-induced release of s-ENTDs was increased. Therefore, the s-ENTD release in the bladder LP appears to be regulated by mechanosensitive bladder afferents that are sensitive to TTX and insensitive to capsaicin. The observation that TTX augmented the distention-induced release of s-ENTDs and consequently accelerated the degradation of extracellular ATP was surprising. Such an effect of TTX would withhold bladder functions that are mediated by ATP released in the LP during bladder filling. In a recent study in mice, TTX significantly reduced bladder afferent firing in response to distention and reduced spinal cord activation during filling, suggesting that TTX-sensitive Na_v_ channels directly regulate the excitability of bladder sensory afferents [42]. The observations made in the present study, however, suggest that TTX may have reduced the excitability of bladder sensory afferents by diminishing the extracellular ATP as a result of the elevated release of s-ENTDs. However, the degradation of ATP was not measured in these studies. The discussion of our results with TTX could be expanded to the potential role of s-ENTDs in bladder nociception. Thus, it could be speculated that effects of Na_v_ channel inhibitors such as lidocaine might also be due to facilitated ATP hydrolysis by s-ENTDs.

The arrival of an action potential to the nerve ending activates voltage-gated Ca^2+^ channels in the neurolemma and induces a Ca^2+^ influx that triggers neurotransmitter release. Therefore, we next examined whether neural inhibition by obstructing the neuronal Ca^2+^ influx would have a similar effect to TTX. Indeed, the distention-induced release of s-ENTDs and, hence, the degradation of eATP were increased in the presence of ω-Ctx GVIA, a selective inhibitor of neuronal Ca^2+^ influx through Ca_v_2.2 (N-type) Ca^2+^ channels [43]. These results confirmed that the activation of sensory afferents during stretching restricts the release of s-ENTDs. ω-Ctx GVIA also increased the spontaneous release of s-ENTDs, suggesting that some Ca_v_2.2 channels might be open in the absence of stretch.

Next, we wanted to determine whether PIEZO1 and PIEZO2—key mechanosensors in urinary function [20,44]—participate in the “controlled” restriction of the mechanosensitive release of s-ENTDs that was revealed in the presence of TTX and ω-Ctx GVIA. PIEZO proteins are the pore-forming subunits of mechanosensitive ion channels that open in response to cell membrane stretching and play a critical role in sensory neuron transduction [45,46]. In the mouse bladder mucosa, PIEZO1 is found expressed in all layers of the urothelium and in subjacent interstitial cells [19], whereas PIEZO2 is found present in a subset of umbrella cells and adjacent urothelial cells [47] and in bladder sensory neurons and DRG [20,48]. Cluster analysis of gene expression patterns in the isolated single DRG neurons of C57BL/6 mice has demonstrated the high expression of PIEZO2 in DRG neurons with characteristics of mechanosensitive Aδ-fiber neurons [49]. Information about the potential role of PIEZO proteins in extracellular purinergic signaling in the bladder is very limited. For example, the downregulation of PIEZO1 resulted in a decreased stretching-induced release of ATP in cultured mouse urothelial cells [50] and interstitial cells in human bladder LP [25]. The deletion of either PIEZO1 or PIEZO2 alone in the mouse bladder urothelium had no significant effect on the release of ATP from the serosal side of the urothelium in response to the distention of the bladder mucosa by filling of the bladder at a supraphysiological flow rate [47]. However, the deletion of both PIEZO1 and PIEZO2 inhibited the ATP release under the same experimental conditions [47], suggesting that the two PIEZO proteins likely work in synergy to regulate the mechanosensitive release of urothelial ATP.

In the present study, we used GsMTx4, a peptide isolated from tarantula venom that alters lipid bilayer mechanics [51] and potently inhibits cationic mechanosensitive channels, including PIEZO1 [52,53] and the transient receptor potential canonical TRPC1 [54] and TRPC6 channels [55,56]. In electrophysiology studies of cells transfected with PIEZO1, the channel showed equal sensitivity to both enantiomers (L- and D-) of GsMTx4 with K_d_~200 nM [57]. D-GsMTx4, in particular, has been suggested to effectively inhibit PIEZO2 channels [58]. In the present study, no significant changes of s-ENTD release in the LP were observed in the presence of each enantiomer alone. The lack of effect of the individual enantiomers of GsMTx4 might be due to the use of subthreshold concentrations (1 µM each) or to the requirement that PIEZO1 and PIEZO2 should be inhibited simultaneously [47,59]. Therefore, in a separate set of experiments, we applied GsMTx4 plus D-GsMTx4 to inhibit both PIEZO1 and PIEZO2. As anticipated, the spontaneous release of s-ENTDs remained unchecked in the presence of the two inhibitors, likely because PIEZO channels were inactive in the absence of stretching. The distention-induced release of s-ENTDs, however, was significantly increased in the presence of the two inhibitors. Treatment of the bladder with the presumed selective PIEZO1 activator Yoda1 [60] had no effect on the release of s-ENTDs. This suggested either that PIEZO1 was not involved in the control of s-ENTD release or that the simultaneous activation of PIEZO1 and PIEZO2 was necessary to limit the release of s-ENTDs. Unfortunately, we could not test this possibility, as, at present, there are no known agonists for PIEZO2 channels. A third possibility is that the effects of GsMTx4 and D-GsMTx4 were mediated not by PIEZO channels but by other proteins that are activated by stretching. As mentioned earlier, GsMtx4 and D-GsMTx4 also inhibit TRPC1 and TRPC6, two members of the TRPC family [55,56] that are expressed in DRG neurons [61] and respond to mechanical stimuli [55,62]. We therefore performed additional experiments to control for this possibility by applying several drugs that affect the functions of TRPC1 and TRPC6. A highly potent inhibitor of TRPC1/4/5, Pico145, had no effect on the distention-induced release of s-ENTDs in the LP. Therefore, it was unlikely that the effects of GsMTx4 plus D-GsMTx4 were caused by inhibition of TRPC1 channels. The TRPC3/6 channel activator GSK 1702934A also had no effect of the s-ENTD release in the LP. The effects of BI 749327, a TRPC6 channel inhibitor, and GSK 2833503A, an inhibitor of TRPC3/6, were more intriguing. The effect of the TRPC6 channel inhibitor was somewhat similar to the effects of PIEZO channel inhibitors in that in both cases, the spontaneous release remained unchanged, whereas the distention-induced degradation of eATP was increased. It should be noted, however, that the effect of GsMTx4 plus D-GsMTx4 was more potent than the effect of GSK 2833503A, with the latter only causing increased levels of eAMP. On the other hand, the inhibition of TRPC3/6 with BI 749327 appeared to increase both the spontaneous and the distention-induced release of s-ENTDs. This effect is different from the effect of combined PIEZO channel inhibitors. Therefore, while TRPC6 channels might participate in the regulation of s-ENTDs in the LP, our data suggest that the effects of GsMTx4 plus D-GsMTx4 were primarily due to the inhibition of PIEZO channels.

These findings also lend additional interpretation to observations made in other studies. For example, PIEZO1 expression is found increased in experimental models of cyclophosphamide-induced cystitis [63] and partial outlet obstruction [64], two conditions that are generally associated with increased bladder excitability. It is logical to assume that the excessive expression of PIEZO proteins could cause the over-sensitization of the urothelium to stretch. It could also be that the higher expression of PIEZO proteins results in greater restriction of the distention-induced release of s-ENTDs elevating the ATP levels at receptor sites. Additionally, “blocked ATP release” in the serosal side of detrusor-free bladder preparations from PIEZO1/2^−/−^ mice [47] might actually reflect accelerated ATP degradation in the absence of PIEZO1 and PIEZO2. However, ATP metabolism was not measured in these studies. Finally, reduced extracellular ATP caused by increased degradation by s-ENTDs may contribute to the signs of bladder underactivity in urothelial [47] and neuronal PIEZO2^−/−^ mice and PIEZO2-deficient humans [20].

The observations made in the present study raise an important question: what is the purpose of having two opposite influences (i.e., increasing and restraining) on the s-ENTD release/ATP hydrolysis in the LP during bladder filling? It is possible that the increased release of s-ENTDs in response to distention during bladder filling prevents excessive (or early) accumulation of ATP at receptor sites by accelerating the ATP hydrolysis. At the same time, it appears that the distention of the bladder wall triggers mechanisms that avert the excessive release of s-ENTDs, preserving optimal levels of ATP. A second model of the s-ENTD-mediated regulation of extracellular purine concentrations might suggest that the processes of increasing and restraining s-ENTD release may be associated with the regulation of the levels of the ATP metabolites ADP and ADO, which have their own biological activities. ADO, in particular, is the dominant purine in the extracellular space in the LP at the end of bladder filling [34]. ADO has been shown to stimulate bladder functions by lowering the threshold for micturition [65] and by enhancing bladder afferent stimulation via the activation of A1 and A2a adenosine receptors in the bladder wall [66]. Therefore, the accelerated degradation of ATP during bladder filling would facilitate the formation of ADO, whereas forces that restrain the release of s-ENTDs during bladder filling would lower the risk of excessive ADO formation at the end of bladder filling. Both models put forward the concept that having optimal extracellular levels of biologically active purines is key for maintaining proper bladder excitability.

Further studies should determine what causes the “controlled inhibition” of s-ENTD release in the LP as the bladder filling progresses. It is possible that the activation of ion channels and sensory afferents in response to bladder wall distention releases a soluble/diffusible factor with the ability to curtail the excessive release of s-ENTDs. Immunohistochemical studies have revealed that bladder sensory afferents contain a number of neuropeptides, such as pituitary adenylate cyclase-activating polypeptide (PACAP), tachykinins, substance P and calcitonin gene-related peptide (CGRP) [15,67,68,69], that can be released from mechanosensitive afferents and in turn can regulate bladder excitability during bladder distention [68,70,71]. Therefore, with the next set of experiments, we investigated whether the pharmacological inhibition of neuropeptide receptors that are known to modulate the activities of mechanosensitive bladder afferents mimics the effects of TTX, ω-Ctx GVIA and PIEZO channel inhibition. A good candidate for the endogenous diffusible factor that performs the “controlled inhibition” of distention-induced s-ENTD release should not alter the constitutive release of s-ENTDs but should diminish the release of s-ENTDs at the end of bladder filling. Therefore, the inhibition of the corresponding neuropeptide receptors should increase the distention-induced release of s-ENTDs but should not alter the spontaneous release of s-ENTDs. First, we examined the effects of the specific antagonist of the NK2 receptor MEN 10376 [72]. Neither the spontaneous nor the distention-induced release of s-ENTDs was altered by MEN 10376, suggesting that the endogenous neurokinin A may not be a strong candidate for the substance that causes the “controlled inhibition” of the distention-induced release of s-ENTDs. The NKA modulation of afferent mechanosensitivity in the bladder appears to be indirect, through the release of NKA from mechanosensitive afferents in the detrusor, which in turn enhances the phasic detrusor contractions/micromotions during physiological bladder filling [22] that increase the activity of afferent nerves and ultimately facilitate the micturition reflex [73]. Therefore, our observations in the presence of the NK2 antagonist may not be surprising if the NK2 receptors that affect the afferent drive are in the detrusor muscle, which is absent in our bladder model. NK2 receptors appear to also be present in capsaicin-sensitive neurons in rat DRG [74]. However, the observations made in the present study suggested that capsaicin-sensitive neurons do not play a significant role in the release of s-ENTDs in the LP.

In contrast to the NK2 receptor inhibitor, the application of PACAP6-38, a potent and competitive PAC1 receptor antagonist [75], mimicked the effects of neural and PIEZO channel inhibition. Thus, in the presence of PACAP6-38, the spontaneous s-ENTD release remained unchecked, whereas the distention-induced release of s-ENTDs in the LP was increased, suggesting that endogenous ligands of the PAC1 receptor may be good candidates for the diffusible substance(s) that restrain the excessive release of s-ENTDs during bladder filling. The endogenous ligands of the PAC1 receptor are PACAP38 and PACAP27; however, PACAP27 is typically 10- to 100-fold less abundant in many tissues [75,76]. In the bladder, PACAP mRNA expression and PACAP immunoreactive sensory fibers are found in the bladder serosa, smooth muscle and suburothelium [77,78]. A recent study has demonstrated that PACAP38 increased bladder afferent nerve activity with distention, and this effect of PACAP38 was blocked by PACAP6-38 [21]. Therefore, PACAP38 should be considered in the search for endogenous mediators that produce the controlled restriction of distention-induced release of s-ENTDs in the LP. Interestingly, mice with a deleted PACAP gene [79] or with the intra-bladder administration of PACAP6-38 [80] showed signs of bladder underactivity, such as an increased bladder capacity, voided volume and intercontraction intervals, as well as large residual volume. Given the results in the present study, it could be speculated that accelerated ATP elimination by PAC1 inhibition was the cause of bladder underactivity in these studies. However, ATP hydrolysis was not evaluated in these experiments. Further studies are warranted to determine peptide and non-peptide substances that might be released in the LP during bladder filling and restrain the mechanosensitive release of s-ENTDs.

**Figure 10:**
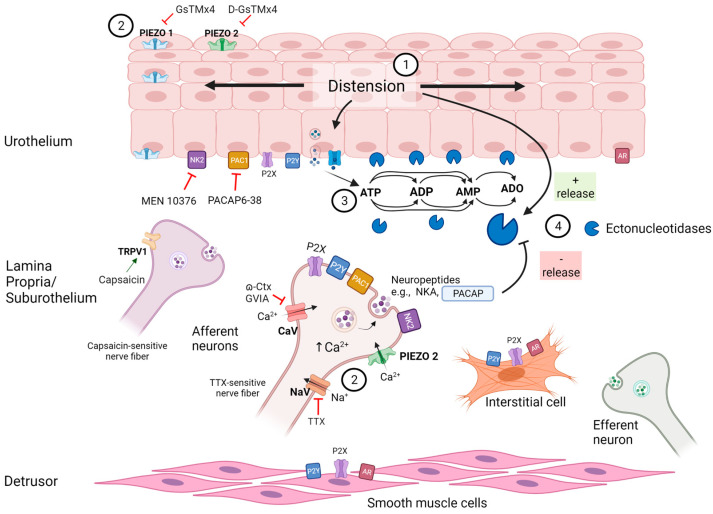
**A model depicting mechanosensitive regulation of s-ENTD release in the bladder lamina propria.** Distention of the bladder wall during bladder filling (**1**) causes activation of PIEZO channels [19,20] and mechanosensitive afferent neurons [15,16,17] (**2**) as well as release of ATP [1,14,17] and of soluble ectonucleotidases (s-ENTDs) [9] from the urothelium into the lamina propria. ATP is then degraded to ADP, AMP and adenosine (ADO) by membrane-bound and s-ENTDs [8,9] (**3**). ATP, ADP and ADO activate P2X, P2Y and adenosine (AR) receptors in various cell types in the LP and detrusor muscle. Simultaneously, activation of PIEZO channels, TTX-sensitive neurons and PAC1 receptors in response to distention restrain the additional release of s-ENTDs (**4**) to avert the excessive degradation of ATP and preserve bioactive purines at effective concentrations at receptor sites. Controlled inhibition of s-ENTD release is likely mediated by substances (e.g., PACAP and other neuropeptides) that are released from sensory neurons and/or urothelium in response to distention during bladder filling. The release of s-ENTDs and consequent extracellular ATP metabolism in the lamina propria is under complex regulation (Figure created with BioRender.com).

In conclusion, the results presented in this study suggest that the activation of neural activity and PIEZO channels by stretching restricts the distention-induced but not the spontaneous release of s-ENTDs and the consequent degradation of ATP in the LP of the mouse bladder (Figure 10). These mechanisms represent a new form of sensory modulation. S-ENTDs appear to be important for the complex purinergic signaling in the LP by controlling the effective concentrations of excitatory and inhibitory purine mediators and preventing hypersensitivity to distention. The bladder LP is equipped to precisely regulate the extent of ATP hydrolysis during bladder filling. Further studies are necessary to determine whether this novel form of mechanosensory modulation is a widespread phenomenon. Understanding the mechanisms of the fine regulation of purine levels at receptor sites may foster the development of novel therapeutic strategies for bladder excitability disorders.

## 4. Materials and Methods

### 4.1. Animals

All animal procedures followed the National Institutes of Health Guide for the Care and Use of Laboratory Animals and were approved by the Institutional Animal Use and Care Committee at the University of Nevada Reno (Protocol number: 20-091077). Male C57BL/6 mice (Jackson Laboratory, JAX stock# 000664), 12–18 weeks of age, were housed in temperature-controlled rooms under 12 h light–dark cycles. The animals received water and standard chow ad libitum. Animals were sedated with isoflurane (AErrene; Baxter, Deerfield, IL, USA) and euthanized by cervical dislocation prior to harvesting the urinary bladders.

### 4.2. Ex Vivo Detrusor-Free Bladder Preparation

Ex vivo bladder preparations with detrusor smooth muscle removed were prepared as described previously [9,34,36,81]. Briefly, the bladder was placed in a dissecting dish filled with oxygenated cold Krebs bicarbonate solution (KBS) and pinned through the serosa to the Sylgard-covered bottom of the dish. KBS was composed of (mM) 118.5 NaCl, 4.2 KCl, 1.2 MgCl_2_, 23.8 NaHCO_3_, 1.2 KH_2_PO_4_, 11.0 dextrose and 1.8 CaCl_2_ (pH 7.4). The fat and connective tissues around the bladder and ureters were cleaned using surgical scissors. Portions of the serosa with the detrusor muscle attached were gently pulled in a lateral direction using fine-tip forceps. Then, segments of the muscle were cut with surgical scissors along the submucosal border of the muscle afar off the urothelium. To preserve all layers of the bladder mucosa, including LP [34], special care was taken to avoid peeling/pulling the detrusor muscle from the urothelium. The detrusor-free preparation was catheterized through the urethra with a PE-20 tubing. The catheter was connected to a syringe pump to fill the bladder with physiological solutions containing drug or vehicle.

### 4.3. General Experimental Protocol

Detrusor-free bladder preparations were placed in 3 mL water-jacketed chambers filled with regular KBS containing drug or vehicle. The solution in the chamber was kept at 37 °C and bubbled with 95% O_2_/5% CO_2_. After equilibration for 30 min, the solution in the chamber was replaced with fresh solution, and the bladder was left empty (nondistended condition) for the time equivalent to filling. Then, 2.9 mL of the bathing solution (designated as extraluminal solution, ELS) was collected and processed further, as described in Section 4.4. Following this step, the bladder was re-equilibrated for 30 min. Then, 3 mL of fresh KBS was added to the chamber and the bladder was filled at 15 µL/min with solution containing drug/vehicle via an infusion pump (Kent Scientific, Torrington, CT, USA) to ~85–90% of bladder capacity, determined at the time of dissection (distended condition) [9,36]. At the end of filling, 2.9 mL of ELS was collected and processed further, as described in Section 4.4. In experiments testing the effects of receptor/channel agonists, the bladder preparations were equilibrated in KBS, and the drug or vehicle was added to the bath during bladder filling (distended condition) or the equivalent time (nondistended condition). In the experiments testing the effects of receptor/channel inhibitors, the drugs were applied to the dissecting solution, the bath and the bladder filling solution to accomplish more complete inhibition of receptors or channels. A complete list of drugs used in this study is shown in Table 1. Only one drug was tested in each bladder preparation.

### 4.4. Preparation of Extraluminal Samples for Measuring Soluble Nucleotidase Activities in the LP

Samples from ELS of nondistended and distended bladder preparations were prepared as described previously [9]. Briefly, the 2.9 mL ELS collected at the end of time equivalent to the time for bladder filling (nondistended condition) or at the end of filling (distended condition) were placed in 4 mL Amicon Ultra centrifugal filter units with 10 kDa molecular weight cut-off (MWCO) pore size (Millipore Sigma, Burlington, MA, USA) to be concentrated by centrifugation. The ELS samples were concentrated to approximately 80–100 µL volume at 4000× *g* for 25 min using SorvallST 40R centrifuge (Thermofisher, Langenselbold, Germany). The concentrated ELS (cELS) were used to detect the degradation of eATP substrate by enzymes that were released in the ELS at rest or during bladder filling.

### 4.5. Time-Course of ATP Hydrolysis in Concentrated ELS of Nondistended and Distended Preparations

Enzymatic reactions to measure the hydrolysis of ATP were carried out at 37 °C, as described previously [9]. Briefly, cELS of either nondistended or distended bladder preparation were placed in 0.6 mL Eppendorf tubes, and the volume in each tube was brought to 200 µL with fresh oxygenated KBS. The substrate eATP was added to the reaction tube at a final concentration of 2 µM. Then, 20 µL samples were taken at 10 s, 2′, 4′, 6′, 8′, 10′, 20′, 30′, 40′ and 60′ from the start of reaction and placed in HPLC inserts containing 180 µL cold citric buffer to stop the reactions and dilute the samples 10-fold. Then, the inserts were placed in HPLC glass vials and stored at −20 °C until HPLC analysis.

### 4.6. Synthesis of 1,N^6^-etheno-ATP Substrate

eATP has ~1,000,000-fold higher fluorescent properties than authentic ATP [82], and when used as substrate, it allows small changes in substrate or product amounts to be detected. The eATP substrate was prepared as described previously [9,34]. Briefly, 0.2 mM ATP (Sigma-Aldrich, St. Louis, MO, USA) were acidified to pH 4.0 with citrate phosphate buffer. 2-Chloroacetaldehyde was added and substrates were heated to 80 °C for 40 min to form 1,N^6^-ethenoderivative of ATP, eATP [82,83].

### 4.7. HPLC Analysis of 1,N^6^-etheno-nucleotides and 1,N^6^-etheno-nucleosides

eATP, eADP, eAMP and eADO in enzyme reaction solutions of nondistended and distended preparations were measured using a reverse-phased gradient Agilent Technologies 1200 liquid chromatography system equipped with a fluorescence detector (Agilent Technologies, Wilmington, DE, USA) as described previously [9,34,82]. Areas of HPLC peaks were calculated and calibrated to individual etheno-derivatized purine standards of eATP, eADP, eAMP and eADO.

### 4.8. Statistical Analyses

Calculations were performed using Excel (Microsoft Corporation, Redmond, WA, USA) and GraphPadPrism version 8.4.2 for Windows (GraphPad Software, San Diego, CA, USA). Values are expressed as mean ± SEM. Comparisons with *p* values < 0.05 were considered statistically significant. Asterisks were used to express statistical significance in figures: * *p* < 0.05, ** *p* < 0.01, *** *p* < 0.001, **** *p* < 0.0001. Two-way analysis of variance (ANOVA) with Sidak’s or Tukey’s post-hoc analysis was used for multiple comparisons.

Parts of this work have been previously presented in abstract form at the sixth annual meeting of the Society for Pelvic Research, Charlotte, NC, 8–10 December 2022.

## Figures and Tables

**Table 1:** Activators and inhibitors of membrane receptors and channels used in this study.

Drug	Mechanism of Action	Working Concentration (µM)	Vehicle	Vendor
**BI-749327**	TRPC6 inhibitor	1	DMSO 0.2%	MedChemExpress, Monmouth Junction, NJ, USA
**Capsaicin**	TRPV1 channel agonist	1	DMSO 0.1%	Tocris Biosciences, Minneapolis, MN, USA
**ω-CtxGVIA**	Ca_v_2.2 (N-type) Ca^2+^ channel inhibitor	0.1	KBS	Tocris Biosciences
**D-GsMTx4**	PIEZO inhibitor	1	KBS	Tocris Biosciences
**GSK 1702934A**	TRPC3/6 activator	3	DMSO 0.2%	Tocris Biosciences
**GSK 2833503A**	TRPC3/6 inhibitor	1	DMSO 0.2%	Tocris Biosciences
**GsMTx4**	PIEZO inhibitor	1	KBS	Tocris Biosciences
**MEN 10376**	NK2 receptor antagonist	10	KBS	Cayman Chemicals, Ann Arbor, MI, USA
**TTX**	Fast Na^+^ channel inhibitor	0.5	KBS	Alomone Labs, Jerusalem, Israel
**PACAP6-38**	PAC1 receptor antagonist	0.3	KBS	Tocris Biosciences
**Pico145**	TRPC1,4,5 inhibitor	1	DMSO 0.2%	MedChemExpress
**Yoda1**	PIEZO1 activator	40	DMSO 0.2%	Tocris Biosciences

## Data Availability

The raw data supporting the conclusion of this article will be made available by the authors upon reasonable request. The data are not publicly available due to privacy.
